# Segmented Filamentous Bacteria – Metabolism Meets Immunity

**DOI:** 10.3389/fmicb.2018.01991

**Published:** 2018-08-24

**Authors:** Grant A. Hedblom, Holly A. Reiland, Matthew J. Sylte, Timothy J. Johnson, David J. Baumler

**Affiliations:** ^1^Department of Food Science and Nutrition, University of Minnesota Twin Cities, Saint Paul, MN, United States; ^2^Food Safety and Enteric Pathogens Research Unit, USDA-ARS National Animal Disease Center, Ames, IA, United States; ^3^Department of Veterinary and Biomedical Sciences, University of Minnesota Twin Cities, Saint Paul, MN, United States; ^4^The Microbial and Plant Genomics Institute, University of Minnesota Twin Cities, Saint Paul, MN, United States; ^5^The Biotechnology Institute, University of Minnesota Twin Cities, Saint Paul, MN, United States

**Keywords:** segmented filamentous bacteria, SFB, *Candidatus* Arthromitus, turkey (*Meleagris gallopavo*), microbiome and immune system

## Abstract

Segmented filamentous bacteria (SFB) are a group of host-adapted, commensal organisms that attach to the ileal epithelium of vertebrate and invertebrate hosts. A genetic relative of the genus *Clostridium*, these morphologically unique bacteria display a replication and differentiation lifecycle initiated by epithelial tissue binding and filamentation. SFB intimately bind to the surface of absorptive intestinal epithelium without inducing an inflammatory response. Rather, their presence impacts the generation of innate and differentiation of acquired immunity, which impact the clearance of extracellular bacterial or fungal pathogens in the gastrointestinal and respiratory tracts. SFB have recently garnered attention due to their role in promoting adaptive and innate immunity in mice and rats through the differentiation and maturation of Th17 cells in the intestinal tract and production of immunoglobulin A (IgA). SFB are the first commensal bacteria identified that impact the maturation and development of Th17 cells in mice. Recently, microbiome studies have revealed the presence of *Candidatus* Arthromitus (occasionally designated as *Candidatus* Savagella), a proposed candidate species of SFB, in higher proportions in higher-performing flocks as compared to matched lower-performing flocks, suggesting that SFB may serve to establish a healthy gut and protect commercial turkeys from pathogens resulting in morbidity and decreased performance. In this review we seek to describe the life cycle, host specificity, and genetic capabilities of SFB, such as bacterial metabolism, and how these factors influence the host immunity and microbiome. Although the role of SFB to induce antigen-specific Th17 cells in poultry is unknown, they may play an important role in modulating the immune response in the intestinal tract to promote resistance against some infectious diseases and promote food-safety. This review demonstrates the importance of studying and further characterizing commensal, host-specific bacteria in food-producing animals and their importance to animal health.

## Introduction

The distal gastrointestinal tract of all animals is colonized by a diverse array of bacterial, fungal, and protozoan species. An animal host maintains a mutualistic relationship with its microbial inhabitants in which the microbes provide protection from pathogenic bacteria through competing for ecological niches and fostering a stable environment for the development of host immunity (Bäckhed et al., 2005). Many vertebrate intestines (such as mice, rats, chickens, humans, and turkeys) harbor commensal organisms named segmented filamentous bacteria (SFB) that bind specifically to the host intestinal epithelium. These organisms are closely related to the genus *Clostridium*, and appear as long, segmented filaments that bind tightly to the host epithelium via a specialized structure (Chase and Erlandsen, 1976). These bacteria were initially detected through microscopic examination of the gastrointestinal epithelium of mice (Davis and Savage, 1974). SFB drew the attention of researchers due to their unique morphology, life cycle and binding location (Schnupf et al., 2013). Since their discovery, a large body of research has been generated to characterize these bacteria and understand their role in the host-microbiome relationship. Through examining mouse and rat models, it has been revealed that these bacteria play an important role in adaptive and innate immunity of the host.

## Morphology and Life Cycle

**FIGURE 1:**
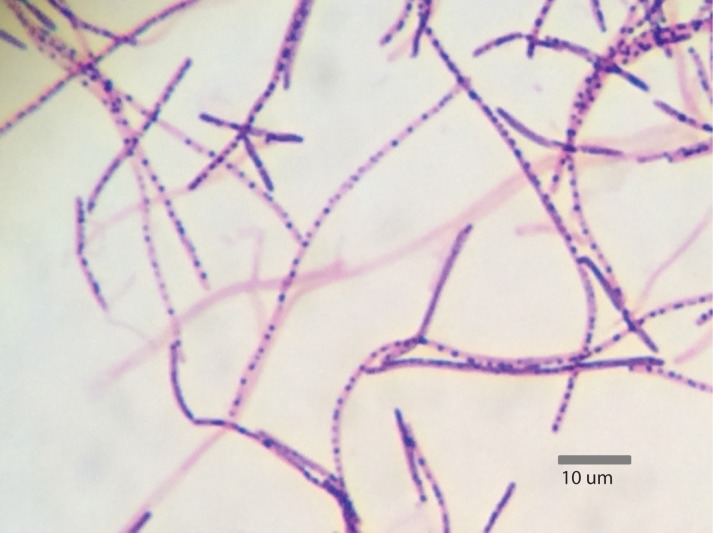
Gram stain of *Candidatus* Arthromitus from turkey ileum containing intrasegmental bodies (1,000× magnification) (Reiland, 2016).

Segmented filamentous bacteria are Gram-positive, spore-forming bacteria that possess the capability to develop into long filaments, which are divided via the production of transverse septa (Ericsson et al., 2014). These bacteria exist in a developmental or vegetative form, which are characterized by the presence of intrasegmental bodies and spores, respectively (**Figure 1**). SFB produce intrasegmental bodies, which can appear as either a spore or a holdfast. The distinction between these morphologies indicates that the holdfast form serves as the vegetative form, while the spore serves as the dormant form (Chase and Erlandsen, 1976).

Segmented filamentous bacteria selectively attach to the ileal epithelium of the host species via the production of rounded, nipple-like projections called holdfasts. The holdfast serves as an anchoring mechanism and point of filament elongation, attaching to host enterocytes in the mucous membrane of the epithelium, without penetrating the host cell wall (Sanford, 1991). This structure leads to the displacement and destruction of the intestinal microvilli surrounding the point of attachment (Snellen and Savage, 1978), and leads to alterations in the electron density of both the host cell plasma membrane and apical cytoplasm (Ericsson et al., 2014). Once attached, actin polymerization occurs directly underneath the holdfast structure and creates a pedestal-like formation similar to the adherence mechanism of *Salmonella enterica* serovar Typhimurium (Jepson et al., 1993; Ericsson et al., 2014). Though this attachment mechanism resembles that of pathogenic bacteria, it does not damage the host cell and causes no inflammatory response in the lamina propria (Caselli et al., 2010). Once attached to the host epithelium, the filament begins to extend from the distal end, releasing additional holdfasts and spores from the maturing filament (Chase and Erlandsen, 1976). The life cycle of an SFB filament is assumed to be about 2–3 days based on the rapid shedding of the intestinal epithelium of rodents, and longitudinal studies have shown that SFB appear in juvenile mice that are around 20 days in age (Davis and Savage, 1974). At this stage of development, SFB proliferate to become a dominant gut microbe and then recede in mature vertebrates to lower levels. During the early stages of colonization, SFB are transiently colonized with rod-shaped bacteria (Schnupf et al., 2013). It is believed that the spread of SFB then occurs via vertical transmission of spores from parents to offspring, as these bacteria are widely considered to be obligate anaerobes and have also appeared in the intestinal tissue of weaning mice (Schnupf et al., 2013). Colonization occurs in mouse and rat hosts at the onset of the weaning process and has been found to be the same in arbitrary human microbiome studies (Ericsson et al., 2014). Studies performed on human subjects ages 0 months to 75 years old revealed that 25% of individuals carry SFB in their gut from ages 0–6 months, 75% carry SFB from ages 7–12 months, and only 6.2% carry SFB from ages 3–75 (Yin et al., 2013). The age-related drop in SFB intestinal carriage may be pharmaceutically reversed. For example, transient feeding mice rapamycin enhanced their lifespan, while dramatically increasing the prevalence of SFB in the small intestine (Bitto et al., 2016). In chickens, SFB colonization peaked at approximately 2 weeks of age, and decreased as they aged to 6 weeks of age. The decrease was inversely proportional to the amount of intestinal IgA present (Liao et al., 2012). It is unknown whether increasing the prevalence of SFB in the intestinal tracts of adults is beneficial, or may result in autoimmunity (e.g., rheumatoid arthritis) (Wu et al., 2010).

Segmented filamentous bacteria spores germinate in the host’s gut to produce teardrop-shaped, single-celled bacteria referred to as intracellular offspring (Schnupf et al., 2013). Amongst intestinal commensals and symbionts, SFB are unique because they penetrate the intestinal mucus layer and intimately associate with host cells without invading the host (Chase and Erlandsen, 1976; Sanford, 1991). It is assumed that the intracellular offspring use flagella to reach the apical surface of polarized epithelium (Kuwahara et al., 2011). Though cellular flagella have not been observed microscopically, an analysis of a SFB genome revealed a full set of chemotaxis and flagellin biosynthesis genes, strongly suggesting the presence of flagella in the early stages of spore maturation (Kuwahara et al., 2011). Intracellular offspring attach to absorptive epithelial cells via their holdfast and induce condensed actin rearrangements underneath the point of attachment while displacing some of the neighboring microvilli structures (Chase and Erlandsen, 1976). Before luminal attachment, the nucleoid region of the intracellular offspring appears condensed, suggesting reduced amounts of transcription. However, when the intracellular offspring attaches to the host, the nucleoid region decondenses and allows for genomic transcription (Chase and Erlandsen, 1976).

Holdfast attachment to enterocytes in the terminal ilea causes the intracellular offspring to increase in size, reaching up to 5 μm in length before bacterial division commences through transverse septum formation (Chase and Erlandsen, 1976). Filaments continue to grow and divide from their distal end, reaching their maximum length of around 50–80 μm (Martin et al., 2009; Schnupf et al., 2013). Once the filament reaches its maximum length, a second round of symmetric division begins from the distal end to divide each original segment in half into secondary undifferentiated cells. These secondary cells range in length from 1 to 1.7 μm, forming segments containing 30–80 cells (Chase and Erlandsen, 1976). After elongation, the filament will often separate from the holdfast segment and enter into the ileum. These secondary segments then undergo differentiation form a mother cell and a daughter cell (Klaasen et al., 1992). Differentiation of these filaments appears to be more pronounced in the presence of slightly aerobic conditions, when oxygen concentrations range from 1 to 2.5% environmental oxygen (Schupf et al., 2015). Once differentiated, the mother cell engulfs and houses the daughter cell, where the daughter cell undergoes division into two intracellular offspring (Klaasen et al., 1992). The intracellular offspring contained within the mother cell are then subject to two fates, dispersal from the filament or sporulation (Davis and Savage, 1974; Klaasen et al., 1992). If favorable growth conditions are present, the septa that separate individual mother cells are degraded to form a tube in which the intracellular offspring are dispersed into the host’s intestinal tract. These released offspring are then allowed to colonize additional host tissue and undergo filamentation and differentiation, thus completing the SFB lifecycle (Martin et al., 2009; Schnupf et al., 2013). When an unfavorable or hostile environment is presented, the two intracellular offspring produce a single spore coat that covers both of the cells. Once coated with the layer of peptidoglycan, the spore matures into a complete endospore inside of the mother cell, and is released from the filament (Martin et al., 2009; Schnupf et al., 2013). These spores lack the ability to colonize the host until favorable environmental conditions, such as appropriate concentrations of oxygen are presented, and shed in the host’s feces. Once shed, the spores can be transmitted to another host via horizontal transmission (Davis and Savage, 1974).

Though it was previously proposed that the entire SFB lifecycle occurred while attached to the host tissue (Chase and Erlandsen, 1976), filaments containing intracellular offspring have not been observed in published TEM and SEM images of filaments attached to enterocytes (Caselli et al., 2010). Alternatively, filaments containing differentiated intracellular offspring only appeared as detached and free-floating, indicating that maturation and differentiation of segments occur independently from host tissues. These filaments are separated from the holdfast segment, which then penetrates the epithelium until it undergoes endocytosis, phagocytosis, or transcytosis (Caselli et al., 2010). The ingestion of the holdfast segment presents a great number of bacterial antigens to antigen presenting cells and lymphocytes contained within the ileal epithelium (Caselli et al., 2010). In rare cases, a small number of segments may remain attached to the holdfast, but these segments often exhibit irregular morphologies and present enlarged intrasegmental junctions (Caselli et al., 2010).

## Characteristics and Metabolism

The genome of a rat-isolated SFB and a number of mice SFB isolates have recently been sequenced and published (Kuwahara et al., 2011; Prakash et al., 2011; Sczesnak et al., 2011; Pamp et al., 2012). These SFB genomes are highly similar but do contain several species-specific genes of unknown function that may be involved in the species-specificity of SFB colonization (Prakash et al., 2011). All sequenced SFB have displayed a small genome size of 1.5–1.62 Mbp, low G+C content (27.9%), and around 1,350–1,400 protein encoding genes (Prakash et al., 2011). SFB possess a highly reduced genome similar to their genetic relatives within the genus *Clostridium* (Ericsson et al., 2014). The biosynthetic pathways of most amino acids, vitamins and cofactors (such as B_1_, B_2_, and B_12_, pyridoxine, nicotinamide, pantothenate, and biotin) are incomplete or absent altogether in SFB genomes (Kuwahara et al., 2011; Sczesnak et al., 2011; Pamp et al., 2012). SFB are also unable to synthesize nucleotides independently; instead they utilize alternative pathways that rely on the uptake of nucleotide bases (Prakash et al., 2011). To obtain nucleotides, amino acids, and peptides from the environment, SFB genomes contains genes encoding two extracellular nucleases as well as a list of proteases and peptidases, 20 of which are membrane associated and 4 to 6 that are thought to be secreted (Kuwahara et al., 2011; Sczesnak et al., 2011). In addition, SFB genomes contain numerous open reading frames (ORFs) thought to encode a large number of transporters and permeases for small molecules and ions (such as amino acids, oligopeptide, dipeptides, manganese, zinc, iron, and phosphate) compared to other organisms with small genomes (Sczesnak et al., 2011). A particularly strong requirement for iron uptake was noted by Sczesnak et al., since six different ORFs for iron transporters are found in the mouse genome as well as three ORFs for ferric iron regulator family proteins (Sczesnak et al., 2011). SFB also have several ORFs for phosphotransferase systems predicted for uptake of sugars such as mannose, cellobiose, mannitol, and fructose (Prakash et al., 2011; Sczesnak et al., 2011). Finally, the SFB genomes contain genes for the non-oxidative pentose phosphate pathway and a complete glycolysis pathway to convert glucose to pyruvate, but are deficient for genes encoding almost all components of the Krebs cycle, which is required for aerobic respiration (Prakash et al., 2011; Sczesnak et al., 2011). However, SFB can tolerate small concentrations of oxygen and counteract oxidative stress, as SFB genomes contain genes predicted for two catalases, a peroxidase (rubrerythrin), and an arginase, which might limit nitric oxide production through catabolism of arginine (Kuwahara et al., 2011; Pamp et al., 2012). These protective mechanisms are likely essential, given the replicative niche of SFB at the surface of the small intestinal epithelium where the oxygen tension is estimated to be around 1.4% (He et al., 1999).

**FIGURE 2:**
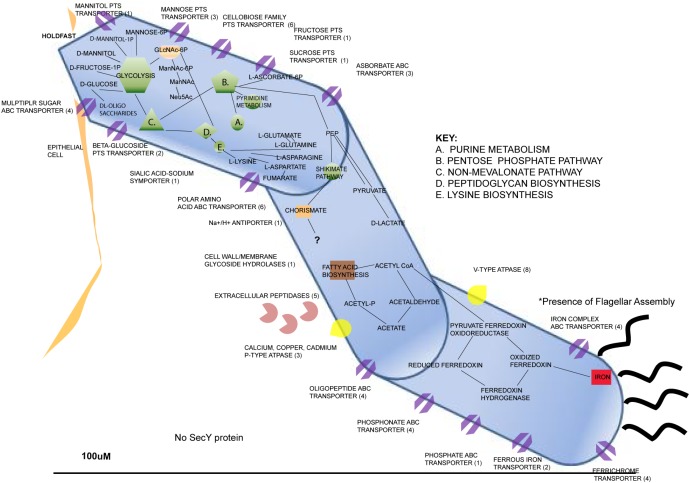
Metabolic features of *Candidatus* Arthromitus through inference of genome contents from annotated genomes through use of Rapid Annotation using Subsystem Technology (Reiland, 2016).

There are several factors that have been discovered about SFB that explain their auxotrophic nature. The genome of SFB isolated from a rat host (Rat-YIT) contains 28 putative genes predicted to encode for proteases and 53 for peptidases along with many other genes through to be involved with sporulation and germination (Prakash et al., 2011). Peroxidase and catalase genes were also found, which explains the potential for SFB to exist in microaerophilic environments (Prakash et al., 2011). The genomes of SFB sequenced from mice and rat hosts revealed several Clustered Regularly Interspaced Palindromic Repeat (CRISPR) loci, which serve as a prokaryotic defense mechanism, indicating that SFB genomes may have had exposure to invading DNA throughout their evolutionary history (Prakash et al., 2011). Flagellar, pilus, and chemotactic genes have been found in SFB genomes that suggest motility, which explains the organism’s ability to penetrate the mucus layer lining of intestinal epithelial cells (Prakash et al., 2011; Schnupf et al., 2013) We have generated a representation of the metabolic and physiological predicted capabilities inferred from the genome contents of publicly sequenced genomes of SFBs (**Figure 2**).

## Host Specificity

**FIGURE 3:**
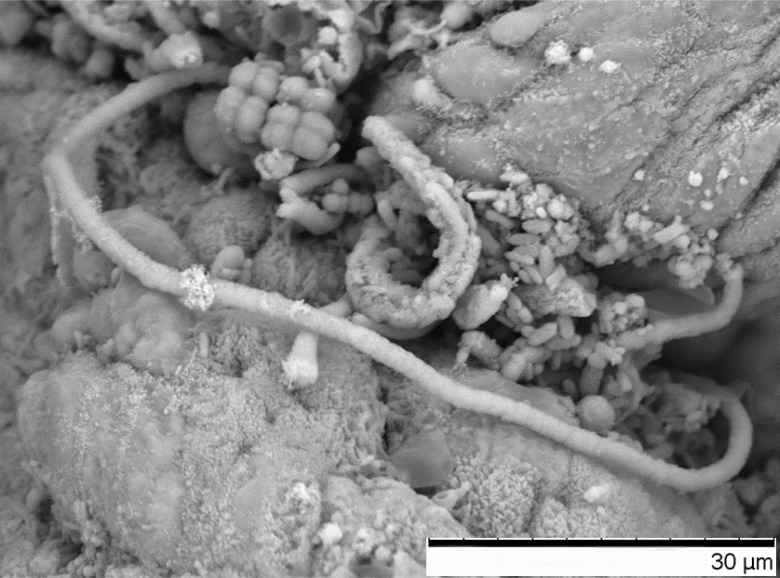
Scanning electron micrograph of a SFB filament attached to epithelium in the cecum of a 28-day old male turkey poult, also pictured are additional unidentified bacteria (2,500× magnification).

An extensive body of work has evidenced the presence of SFB in a large number of animal species, such as horses, cattle, pigs, turkeys, chickens and even humans (Ericsson et al., 2014). In nearly all of the mammalian species studied for the presence of SFB, the bacteria selectively colonize the ileum of the host, with the exception of fish species that lack a well-defined ileum (Ericsson et al., 2014). In poultry, SFB colonizes ileum (**Figure 1**), the cecal tonsil or cecum (**Figure 3**; Rahimi et al., 2009; Bohorquez et al., 2011; Liao et al., 2012). Attempts to colonize an animal species with SFB from another species have been unsuccessful. When germ-free mice and rats were inoculated with ileal homogenates containing SFB from both species, animals became colonized with SFB from their own species, indicating that SFB are host-specific and host selective (Tannock et al., 1984). Host-specificity of SFB may be due to differences in the sequences of flagella genes *fliC3* and *flicC4*, which show greater variability than *fliC1* and *fliC2* (Chen et al., 2017). It is not known with which host proteins the SFB flagella proteins interact to become adherent, however, SFB flagellar proteins may induce Th17 cells by signaling through Toll-like receptor 5 (TLR5) in a subset of CD11c^hi^CD11b^hi^ intestinal dendritic cells (Uematsu and Akira, 2009). For most animal species, holdfast cells have the capability to attach to goblet cells, M-cells, absorptive enterocytes, and cellular junctions of the ileal epithelium (Meyerholtz et al., 2002), whereas less is known for poultry. Species to species variance exists in the preferred cell of attachment. For example, SFB in rats and pigs attach to follicle-associated epithelial cells (over Peyer’s Patches) and absorptive ileal villi (Tannock et al., 1984). In mice and horse, attachment occurs primarily to follicle-associated epithelial cells and mainly to absorptive ileal villi for rabbits, cattle and canines (Pamp et al., 2012). SFB are unique amongst intestinal commensals and symbionts because they penetrate the intestinal mucus layer and intimately associate with host cells, but do not invade the host (Sanford, 1991). The exact mechanism of host specificity remains unclear, but it is believed that initial binding of the holdfast segment to the host epithelium serves as a ligand-receptor interaction, triggering a response from the host (Kuwahara et al., 2011). SFB binding elicits actin polymerization and condensation at the point of attachment (Chase and Erlandsen, 1976), suggesting a specific host response. Host specificity of SFB suggests that these bacteria have coevolved with their hosts to promote the commensal relationship that exists between the two species.

## Effect on Host Microbiome

Though initially considered to be common commensal members of the host microbiota, recent research suggests that SFB serve an important role in modulating host microbiome and immunity. SFB are unique amongst intestinal commensals and symbionts because they penetrate the intestinal mucus layer and intimately associate with host cells without invading the host (Chase and Erlandsen, 1976; Lee et al., 2011; Mortha et al., 2014; Atarashi et al., 2015; Bunker et al., 2015; Farkas et al., 2015; Furusawa et al., 2015; Sadler et al., 2017). The holdfast binding of SFB to the ileal mucosa does not elicit a strong inflammatory response (Klaasen et al., 1991). Several interactions between the host and SFB have been investigated, indicating almost entirely positive associations between the SFB and the host, with rare exceptions. Intestinal colonization of SFB in Rainbow trout may produce a fatal disease called Rainbow Trout Gastroenteritis (RTGE), where SFB expand to large numbers in the intestinal tract and cause enterocytes to detach (Del-Pozo et al., 2010). SFB were not always present near to all intestinal RTGE lesions, suggesting that SFB affects intestinal barrier function and other bacteria may not be crucial for producing RTGE lesions. Experimental infection of susceptible Rainbow trout stock with feces from RTGE animals induced colonization and characteristic histopathological lesions containing SFB and an unidentified Gram-negative coccus, suggesting that SFB, in part, may be the etiological cause of RTGE (McCarthy et al., 2016). Recent advances have identified different methods to culture SFB *in vitro*, accelerating research to study the interaction of SFB with different members of the intestinal microbiota (Ericsson et al., 2015; Schupf et al., 2015).

## Effect on Host Immunity

A large amount of research has demonstrated the indispensable role that SFB play in the maturation of the host gut immune barrier, inducing both innate and adaptive immune responses (Ivanov et al., 2009; Sonnenberg et al., 2011; Mortha et al., 2014; Atarashi et al., 2015; Bunker et al., 2015; Farkas et al., 2015; Furusawa et al., 2015; Edelblum et al., 2017; Schnupf et al., 2017). Immune modulation of SFB may extend beyond the intestinal tract in the blood, or to other mucosal barriers (McAleer et al., 2016). Mice colonized with SFB were more resistant to sepsis secondary to experimental cecal ligation and puncture injury (Cabrera-Perez et al., 2016). Oral vancomycin treatment to mice diminished the number of intestinal Gram-positive bacteria (including SFB), which negatively impacted anti-fungal Th17 immunity in the respiratory tract (McAleer et al., 2016). These data suggest that the composition of intestinal microbiota, especially SFB, is vital for impacting immunity to bacterial and fungal pathogens beyond the intestinal tract.

Segmented filamentous bacteria are best known for their ability to induce the differentiation of naïve CD4^+^ T cells to form antigen-specific Th17 CD4^+^ cells (Schnupf et al., 2013) in the terminal ileum of mice (Farkas et al., 2015). Ivanov et al. first demonstrated that conventionally raised B6 mice purchased form Taconic Farms were highly colonized with SFB; these bacteria were absent from conventional raised mice purchased from the Jackson Laboratory (Ivanov et al., 2009). Introduction of SFB to B6 mice from the Jackson Laboratory induced IL-17A and IL-22 production from intestinal CD4^+^ T cells, which became refractory to colitis induced by the intestinal pathogen *Citrobacter rodentium* (Ivanov et al., 2009). IL-22 is a cytokine that enhances production of antimicrobial peptides from intestinal epithelial cell and prevent bacterial pathogens from inducing attaching and effacing lesions (Schupf et al., 2015). SFB are not the only bacteria capable of inducing Th17 CD4+ T cells in the intestinal tract of animals. Virulent Shiga-toxin producing *E. coli* (O157) and *Citrobacter rodentium* induced a Th17 response in the murine intestinal tract, and was dependent on bacterial adherence to host cells (Atarashi et al., 2015), as well as the commensal *Bifidobacterium adolescentis* (Tan et al., 2016). Th17 cells are a subset of CD4^+^ T cells that are distinguished by the expression of T cell receptor CD3, nuclear transcription factor RAR-related orphan receptor gamma T (RORγt) and production of interleukins IL-17A, IL-17F, IL-21, and IL-22 (Schnupf et al., 2017). Other immune cells present in the intestinal lamina propria are capable of secreting IL-17A and IL-22 or express RORγt [e.g., innate lymphoid cells type 3 (ILC3) and lymphoid tissue inducer-like cells (LTi)], but these cells lack expression of CD3 (Sonnenberg et al., 2011; Mortha et al., 2014). However, ILC3 and LTi are not dependent on SFB for their induction. In the intestinal epithelium, IL-17A and IL-17F help to modulate neutrophil chemotaxis through producing CXCL chemokines via binding interactions with IL-17 receptors IL-17RA and IL-17RC. IL-17A and IL-17F additionally aid in regulating the activation and differentiation of host neutrophils, and stimulate the production of host-defense peptides (Schnupf et al., 2017). Systemic depletion of neutrophils in mice caused increased production of IL-17A and ileal SFB colonization (Flannigan et al., 2017). Thus, neutrophil recruitment may lessen IL-17A and chemokine production, and serve as a negative feedback loop to limit SFB colonization. The regulation of these immune-stimulatory compounds and cell types is essential in combatting intestinal colonization and infection from microorganisms. Th17 cells provide colonization resistance to other pathogenic bacteria present at mucosal barriers, such as *Escherichia coli* in the intestinal tract (Zheng et al., 2008; Edelblum et al., 2017) and respiratory fungi (McAleer et al., 2016).

In newborn or germ-free mice, the presence of Th17 cells in the lamina propria is rare, appearing only after colonization by microbes (Gaboriau-Routhiau et al., 2009). The role of SFB in Th17 cell production was initially demonstrated when mice were inoculated with mouse, rat, and human microbiota containing bacterial spores similar to that of the genus *Clostridium*. Only the experimental mice inoculated with a mouse-derived bacteria were shown to produce Th17 cells in response to colonization. Mice colonized with rat- and human-derived bacteria produced much less of a Th17 response when compared to the mouse-derived microbiome treatment, indicating host-specific bacteria (such as SFB) as the causative agent of the immune response (Gaboriau-Routhiau et al., 2009; Chung et al., 2012). This association was also confirmed when 16S rRNA sequencing was performed on the gut microbiome of mice presenting ileal Th17 cells, revealing the presence of SFB (Ivanov et al., 2009). In experiments testing the reactivity of mouse lamina propria against a SFB expression library, two proteins of unknown function elicited a Th17 cell response (Yang et al., 2014). It was predicted that these unknown proteins may serve as cell surface proteins, potentially elucidating the role that SFB attachment may serve in stimulating host immunity (Yang et al., 2014). Proteins from SFB, secreted or bacterial-associated, are believed to interact with host cells and modulate immunity include ADP-ribosyltransferases and a myosin-cross reactive antigen (Pamp et al., 2012). The exact antigen presenting cell responsible for immune modulation by SBF is controversial, but it appears that SFB antigens presented to both intestinal macrophages (Panea et al., 2015) or CD103^+^ intestinal dendritic cells (Goto et al., 2014) are involved. Because of the intimal relationship of SFB with intestinal epithelium, it is possible that metabolites from SFB may also impact the differentiation of Th17 cells. Intestinal macrophages, and not intestinal dendritic cells, appear to be vital for generating SFB-specific Th17 responses in the murine ileum (Panea et al., 2015). Analysis of the T cell receptor repertoire of Th17 cells recognize peptide antigens produced by SFB (Yang et al., 2014). The addition of the Th1-indcing bacterial pathogen *Listeria monocytogenes* failed to impact induction of Th17 cells in SFB colonized mice (Yang et al., 2014), suggesting that the match of T-cell effector function with antigen specificity is driven by the type of bacteria that produce the antigen. Th17 cell differentiation is additionally mediated through the production of serum amyloid A (SAA) and reactive oxygen species (ROS) produced in response to SFB binding. Production of SAA in the host epithelium is initiated by SFB binding and subsequent actin rearrangements, leading to a signal amplification via IL-22 and ILC3, both of which aid in Th17 cell differentiation (Schnupf et al., 2017). SAA also stimulates intestinal antigen presenting cells to secrete IL-23, which assists in Th17 activation and survival (Schnupf et al., 2017), but IL-23 also has as an antagonistic effect on development of Th17 immunity (Shih et al., 2014). ROS produced as a consequence of SFB binding to enterocytes helps to create a chemical environment that promotes Th17 differentiation, as demonstrated in mice treated with ROS scavenging compounds having lower amounts of Th17 cells *in vivo* (Atarashi et al., 2015). SFB flagellar proteins may induce Th17 cells by signaling through TLR5 in a subset of CD11c^hi^CD11b^hi^ intestinal dendritic cells (Uematsu and Akira, 2009). The flagellar binding motifs that are targeted by TLR5 appear to be highly conserved in SFB and is nearly absent from other similar *Clostridium* species, suggesting a specific role for SFB to modulate Th17 immunity (Prakash et al., 2011). Although SFB-induced Th17 immunity may benefit the animal host, there are long-term consequences. SFB-induced Th17 immunity is linked to the development of autoimmunity in susceptible breeds of mice (Lee et al., 2011; Yang et al., 2014; Teng et al., 2016) by inducing differentiation and egress of T follicular cells from Peyer’s patches (Teng et al., 2016). It is unknown whether natural colonization by SFB in poultry is capable of promoting autoimmunity, but it must be considered if SFB, or its antigens are utilized as immunomodulators for food-producing animals.

Segmented filamentous bacteria mono-associated mice display rapid growth and development of Peyer’s patches, and SFB can also stimulate the formation of lymphoid follicles and tertiary lymphoid tissues in the host (Lecuyer et al., 2014). This activation of the host’s intestinal immunity causes a drastic increase in fecal concentrations of secretory Immunoglobulin A (sIgA), as the number and activity of IgA secreting B-cells rises (Klaasen et al., 1991). Germ-free mice monoassociated with SFB triggers the production of IgA serum levels equivalent to that of specific pathogen free, SFB-negative mice (Klaasen et al., 1993). The expansion and stimulation of germinal centers present in Peyer’s patches is not entirely unique to SFB and has been seen to occur in other commensal bacteria such as *Morganella morganii* and *Bacteroides distasonis*, however, the response from SFB is much greater than these other organisms (Schnupf et al., 2013). Both T-cell dependent (B-2 cell) and T-cell independent (B-1 cell) production of sIgA occurs in SFB mono-associated mice (Schupf et al., 2015). The amount of SFB-specific IgA produced by the host in response to bacterial colonization is as high as 1.4% of the total IgA of the organism (Talham et al., 1999). IgA transmitted by nursing mice to suckling pups has been shown to inhibit SFB colonization, and only after weaning do SFB populations begin to increase, coinciding with the time in which SFB colonization is typically recognized in mice (Jiang et al., 2001). The induction of sIgA by SFB may serve as a negative feedback mechanism to prevent overcolonization by SFB and dysbiosis in older animals (Ohashi et al., 2010; Liao et al., 2012).

## Segmented Filamentous Bacteria in Turkeys

Light Turkey Syndrome (LTS) is growing problem facing commercial turkey production in the United States. LTS is a condition in which turkey flocks fail to meet their genetic potential weight, yielding birds that are 4–5 pounds below the industry standard for average flock weight (Danzeisen et al., 2013). Birds affected by LTS display symptoms similar to poult enteritis complex (PEC), a disease in which birds experience weight loss, diarrhea, lethargy, and depression (Mor et al., 2013). However, LTS is dissimilar to PEC in that birds do not experience watery and pale intestinal contents or distended ceca, indicating differences between the syndromes (Morishita et al., 1992). The causative agent of PEC is suspected to be microbial in nature, as inoculation of healthy birds with fecal homogenates derived from birds experiencing PEC produced light weight poults when compared to un-inoculated birds, but a single responsible microbe has yet to be determined (Mor et al., 2013). Similarly, inoculating healthy birds with fecal homogenates derived from turkeys with LTS produced birds that were lighter than the control groups (Mor et al., 2013). The two conditions are not dependent on each other, as LTS can occur in the absence of PEC (Danzeisen et al., 2013). There exist a number of potential factors that may lead to the development of LTS, such as colonization by pathogenic bacteria, viral infection, stunting of immune system development, inhibited nutrient absorption, and alterations to gut microbiome (Danzeisen et al., 2013). Typically, LTS/PES affects birds less than 3 weeks of age (Morishita et al., 1992). A higher number of different pathogenic organisms are found in these younger birds than in birds aged 4–9 weeks. Virus strains such as astrovirus, reovirus, and rotavirus types were detected in the host, and are not associated with poult mortality. Coronavirus, which is commonly associated with mortality, was not detected in LTS/PES poults (Morishita et al., 1992).

In an attempt to understand the microbial basis of LTS, Danzeisen et al. performed 16S rRNA microbiome analysis of low-performing and high-performing (based upon flock weights) flocks to determine the role of microbial succession in promoting digestive health Samples were sequenced to discern the presence and abundance of dominant OTUs present in higher-performing flocks as compared to lower-performing flocks (Danzeisen et al., 2013). After analysis it was determined that at the age of 2–3 weeks, higher-performing turkey flocks harbored significantly higher proportions of *Clostridium bartlettii* and *Candidatus* division Arthromitus, a SFB (Danzeisen et al., 2013). Previous studies regarding SFB colonization of poultry have identified SFB as a causative agent in intestinal disease (Goodwin et al., 1991), but SFB was later ruled out as a causative agent (Sell et al., 1992). Also, SFB belong to several microbial taxa and are not considered a homogeneous group (Thompson et al., 2013). As demonstrated in mice and rat models, SFB have been proven to be potent stimulators of host immunity and ileal health. Though the role of SFB (in particular *Candidatus* Arthromitus) in the digestive health of turkeys is not quite understood, the evidence provided by Danzeisen et al. suggests that epithelial binding of these bacteria may promote early digestive health (Danzeisen et al., 2013). The potential for *Candidatus* Arthromitus to serve as an immunostimulatory probiotic makes it an organism of great interest to poultry researchers, as the turkey production industry is in need of alternatives to promote animal health in this age of restricted use of antibiotics in food-producing animals and increasing antimicrobial resistance.

## Conclusion

Since their initial characterization in the 1970s, SFB have transitioned from being considered an interesting and unique member of the gut microbiome with a unique morphology, to serving as a model organism to study immunomodulatory symbiotic bacteria and their effects on the host. The host-specific binding mechanism employed by these bacteria to attach to ileal epithelium is similar to that of enteric pathogens. Unlike enteric pathogens, SFB do not harm the host epithelium and instead live in a commensal, if not mutualistic manner. Intimate binding to the host mucosal epithelium allows SFB to receive nutrients from the host, satisfying their auxotrophic requirements, while delivering antigens to the host. Epithelial binding also initiates several immune responses from the host. As demonstrated in mice and rat models, SFB have been shown to stimulate the maturation of the host’s Th17 and IgA responses, improving the ability of the host to protect against invading pathogens. Additionally, SFB compete with other members of the intestinal microbiota by modulating access to nutrients and occupying available ecological niches. The fitness-bolstering effects produced by SFB in mouse models are well-understood, but little is known about the roles these bacteria play in the other vertebrate animals. It has been suggested through microbiome analyses of turkeys that SFB, specifically *Candidatus* Arthromitus, may provide a protective role in preventing the onset of the enteric condition LTS, the cause of which is not well understood. The role of SFB in turkeys must be better elucidated to determine the beneficial effects these bacteria have in disease prevention and in ileal health. Developing an understanding the role that commensal microorganisms, like SFB, play in the overall function of the gut microbiome will aid in our understanding the interplay between microbiome and host, providing insights into digestive health and the development of immunity.

## Author Contributions

HR and GH contributed equally to the writing of the manuscript. MS, TJ, and DB also helped to write the manuscript.

## Conflict of Interest Statement

The authors declare that the research was conducted in the absence of any commercial or financial relationships that could be construed as a potential conflict of interest.
